# Origin of ultradian pulsatility in the hypothalamic–pituitary–adrenal axis

**DOI:** 10.1098/rspb.2009.2148

**Published:** 2010-02-03

**Authors:** Jamie J. Walker, John R. Terry, Stafford L. Lightman

**Affiliations:** 1 Bristol Centre for Applied Nonlinear Mathematics, Department of Engineering Mathematics, University of Bristol, Bristol, UK; 2 Henry Wellcome Laboratories for Integrative Neuroscience and Endocrinology, University of Bristol, Bristol, UK

**Keywords:** neuroendocrine regulation, glucocorticoid hormones, ultradian pulsatility, mathematical modelling, numerical continuation

## Abstract

The hypothalamic–pituitary–adrenal (HPA) axis is a neuroendocrine system that regulates the circulating levels of vital glucocorticoid hormones. The activity of the HPA axis is characterized not only by a classic circadian rhythm, but also by an ultradian pattern of discrete pulsatile release of glucocorticoids. A number of psychiatric and metabolic diseases are associated with changes in glucocorticoid pulsatility, and it is now clear that glucocorticoid responsive genes respond to these rapid fluctuations in a biologically meaningful way. Theoretical modelling has enabled us to identify and explore potential mechanisms underlying the ultradian activity in this axis, which to date have not been identified successfully. We demonstrate that the combination of delay with feed-forward and feedback loops in the pituitary–adrenal system is sufficient to give rise to ultradian pulsatility in the absence of an ultradian source from a supra-pituitary site. Moreover, our model enables us to predict the different patterns of glucocorticoid release mediated by changes in hypophysial-portal corticotrophin-releasing hormone levels, with results that parallel our experimental *in vivo* data.

## 1. Introduction

Frequency of coding of intercellular signals is a well-accepted mode of communication between neurons. More than this, however, it is actually a common mechanism of communication across a broad range of both inter- and even intra-cellular systems (Goldbeter 1996). Even an organism as primitive as the slime mould (*Dictyostelium discoideum*) only aggregates in response to external pulses of cyclic AMP delivered with a periodicity of 5 minutes and not to constant stimuli or frequencies greater than every 2 minutes (Darmon *et al*. 1975).

In mammals, the endocrine system is one of the major signalling systems to use frequency encoding. In addition to the vital metabolic hormone insulin (Lang *et al*. 1979), the best described endocrine systems that signal through ultradian rhythms are found in the hypothalamic–pituitary neuroendocrine pathways. Pulsatile gonadotropin-releasing hormone (GnRH) release results in the concordant release of LH pulses from the pituitary (Belchetz *et al*. 1978; Clarke 2002), while modulation of GnRH pulse frequency can produce differential LH and FSH secretion (Wildt *et al*. 1981) via regulation of LH beta and FSH beta mRNA expression (Papavasiliou *et al*. 1986). Interactions between hypothalamic somatostatin and growth-hormone-releasing hormone neuronal systems result in episodic release of growth hormone (GH) (Plotsky & Vale 1985), which is in turn an important factor in mediating GH-dependent gene expression (Waxman *et al*. 1995).

**Figure 1.**
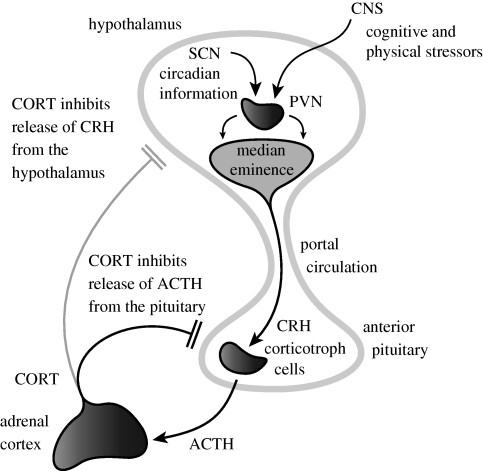
Regulation of HPA axis activity. The hypothalamic PVN receives circadian inputs from the SCN and homeostatic/stress inputs from the brain stem and limbic areas. The PVN projects to the median eminence where it releases CRH into the portal circulation. This passes to corticotrophs in the anterior pituitary which release ACTH from pre-formed granules into the venous circulation. This ACTH reaches the adrenal cortex where it activates the synthesis and secretion of CORTisol (in man) or CORTicosterone (in the rodent). CORT in turn feeds back to inhibit the release of ACTH from the anterior pituitary, and to a lesser extent, CRH from the hypothalamus.

Another system that is characterized by an ultradian rhythm is the hypothalamic–pituitary–adrenal (HPA) axis (figure 1). This stress-responsive neuroendocrine system is extremely well adapted to respond to homeostatic challenge. The HPA axis governs the circulating levels of vital glucocorticoid hormones (CORT), which in turn have major regulatory effects on the cardiovascular, metabolic, cognitive and immunological state of the animal (Chrousos 1995; de Kloet *et al*. 2005; McEwen 2007). The central regulator of this axis—the paraventricular nucleus (PVN) of the hypothalamus—is a major relay for afferent information from limbic areas of the central nervous system that can detect cognitive or emotional stressors, and also from brain stem structures that detect more physical stressors such as inflammation or hypotension (Ulrich-Lai & Herman 2009). The PVN also receives a major input from the hypothalamic suprachiasmatic nucleus (SCN) that coordinates the body's circadian rhythms (Reppert & Weaver 2002). The corticotrophin-releasing hormone (CRH) and arginine vasopressin (AVP) containing parvocellular neurons in the PVN project to the median eminence of the hypothalamus from where they release CRH and AVP into the hypothalamic–pituitary portal circulation (Engler *et al*. 1989; Ixart *et al*. 1991). The CRH and AVP pass along this vascular route to access their receptors on corticotroph cells in the anterior pituitary. These cells in turn are activated by occupation of their CRH and AVP receptors to release corticotrophin (ACTH) into the general circulation through which it accesses the glucocorticoid-secreting cells in the cortex of the adrenal gland. It is these cells that synthesize and release the final product of HPA activation—the glucocorticoid hormones. The final link in this circuit is that physiological levels of glucocorticoid hormones themselves feedback in a negative manner predominantly on the pituitary gland—but also at the level of the PVN and hippocampus—to inhibit further release of ACTH (Jones *et al*. 1977; Dallman *et al*. 1987).

**Figure 2.**
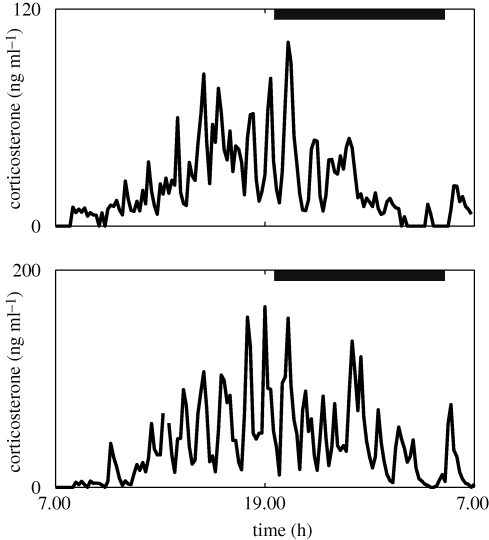
Experimental data demonstrating the ultradian glucocorticoid rhythm underlying the classic circadian profile. Levels of blood corticosterone were recorded over a 24 h period in two individual male Sprague–Dawley rats. Blood samples were collected every 10 min using an automated blood sampling system. Grey bars indicate the dark phase (19.15–05.15 h). Adapted from Spiga *et al*. (2007).

The HPA axis has a unique pattern of activity. Levels are low during the periods of sleep inactivity and increase in anticipation of waking, peaking in the morning in man (Weitzman *et al*. 1971) and evening in the rodent (Dallman *et al*. 1978), with the resultant classic circadian rhythm. This rhythm, however, is not made up of a simple smooth change in hormone levels over the 24 hours. The circadian changes of glucocorticoids are a result of changes in the activity of an underlying ultradian rhythm (Veldhuis *et al*. 1989; Jasper & Engeland 1991; Windle *et al*. 1998*a*; Spiga *et al*. 2007). Glucocorticoids are actually released from the adrenal gland in discrete pulses that result in rapidly changing levels of hormone, both in the blood and within the tissues (figure 2). It is in fact the changes in pulse amplitude, and to a lesser extent frequency, that make up the circadian rhythm (Lui *et al*. 1987; Iranmanesh *et al*. 1989; Veldhuis *et al*. 1989, 1990; Windle *et al*. 1998*b*) and the changes of HPA activity that occur in response to altered physiological and pathological conditions. This pulsatility of glucocorticoid secretion is also an important factor in determining the responsivity of the HPA axis to stress (Windle *et al*. 1998*a*; Lightman *et al*. 2008) and the transcriptional responses of glucocorticoid responsive genes (Stavreva *et al*. 2009).

We have good evidence that the SCN determines the circadian activity of the HPA axis by modulating the inhibitory gain to the PVN; however, we have no idea of the mechanism responsible for the regulation of ultradian activity. Although it is often simply presumed that there must be some sort of hypothalamic pulse generator, there is no good evidence for its existence. The only supportive data come from studies with cultured explants of the macaque hypothalamus (Mershon *et al*. 1992) and from rat median eminence (Ixart *et al*. 1991) that show episodic release of CRH. The relevance of this is unclear, particularly in the absence of cyclic feedback of inhibitory signals from circulating glucocorticoids that are now known to have rapid inhibitory effects even on basal HPA activity (Atkinson *et al*. 2008). Indeed, there is good evidence for the lack of importance of a pulsatile CRH signal for this ultradian rhythm from studies in sheep that have had surgical disconnection of the hypothalamus from the pituitary. These animals still maintain pulsatile cortisol secretion, despite their loss of a normal response to stress (Engler *et al*. 1990). This clearly shows that even in the absence of the stress-activatable hypothalamic input, the ultradian rhythm of cortisol secretion is maintained.

Understanding the mechanisms underlying ultradian HPA activity is very important. It is becoming increasingly clear that glucocorticoid responsive genes respond to these rapid fluctuations in a biologically meaningful way (Stavreva *et al*. 2009) and that a number of psychiatric and metabolic diseases are associated with changes in cortisol pulsatility (Young *et al*. 2004, 2007). Motivated by recent accounts of feed-forward and feedback loops supporting robust oscillations in a number of biological contexts (Stricker *et al*. 2008; Tsai *et al*. 2008; Tigges *et al*. 2009), we hypothesized that the pituitary–adrenal system (which contains a positive delayed feed-forward connection between ACTH and CORT (Papaikonomou 1977), as well as negative nonlinear feedback of CORT on ACTH mediated by the glucocorticoid receptor (GR) (Drouin *et al*. 1992)) could support ultradian oscillations in the absence of a hypothalamic pulse generator. To address this hypothesis, we considered a deterministic theoretical model characterizing the principal interactions between the anterior pituitary and the adrenal cortex (see figure 1, and also figure S7 in the electronic supplementary material). We employed a powerful mathematical technique called numerical continuation (Kuznetsov 1995; Engelborghs *et al*. 2001; Krauskopf *et al*. 2007)—enabling us to systematically characterize how the behaviour of the system depends on the parameters of the system (see the electronic supplementary material for more details)—to explain the mechanisms giving rise to natural oscillatory rhythms in the HPA axis.

## 2. Results and discussion

The aim of model development for the HPA axis was to elucidate whether—using biologically motivated approximations of each of the main compartments of the axis—the system could support ultradian glucocorticoid fluctuations in a similar manner to those observed experimentally, and to explore mechanisms by which these could occur. For this purpose, we adapted a recently proposed model (Gupta *et al*. 2007) using ordinary differential equations (ODEs) that provided a compromise between analytical tractability and biological plausibility. This approach allowed for the integration of experimentally determined parameter values (where known), while permitting a theoretical analysis using a simplified model with the potential for refinement using experimental data. One of the key assumptions made during the modelling process was that the rapid inhibition of hypothalamic CRH by glucocorticoids is not an important factor. This relates back to the fact that the anterior pituitary is the major site for glucocorticoid feedback (Keller-Wood & Dallman 1984) and the relatively slow effect of glucocorticoids on CRH gene transcription (Ma *et al*. 1997). Specifically, the model uses linear mass action kinetics to describe the dynamic levels of ACTH, GR and CORT, and incorporates a delay term to account for the well-known delay in the CORT response to ACTH that results from the lack of releasable pools of CORT and the need to synthesize the hormone for release (see the electronic supplementary material for more details).

**Figure 3.**
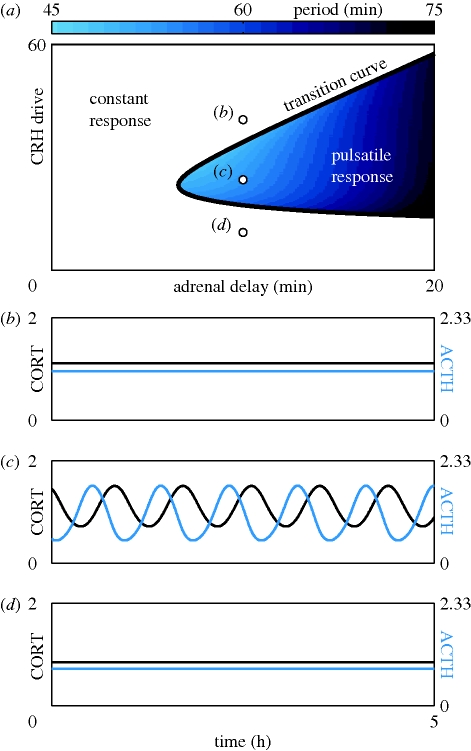
Response of the pituitary–adrenal system to constant CRH drive. Units of all hormone levels are arbitrary. (*a*) Different combinations of constant CRH drive and delay can lead to two qualitatively different responses. On one side of the transition curve, the pituitary–adrenal system responds with constant levels in ACTH and CORT. On the other side of the transition curve, the pituitary–adrenal system responds with pulsatile fluctuations in the levels of ACTH and CORT, despite the fact that the CRH drive is constant. In the region of pulsatile response, the frequency of the pulses is indicated by the colour bar. (*b*–*d*) Model predictions for ACTH (blue) and CORT (black). Each time series was computed with the same delay (10 min), but different levels of constant CRH drive, as indicated by the three points in (*a*).

**Figure 4.**
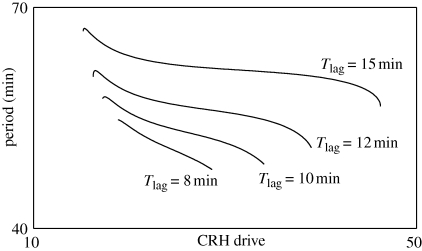
Period of CORT pulses inside the pulsatile region. Period of ultradian CORT rhythm computed for different values of the adrenal delay *T*_lag_ (min) and different levels of CRH drive (arb. units). For all four values of the delay, we observe ultradian pulses with a physiological period. See also the colour bar in figure 3*a*.

Using numerical simulations and continuation methods (see the electronic supplementary material for more details), we determined a range of values in both CRH drive and delay for which ultradian activity was observed in ACTH and CORT (figure 3*a*,*c*). It is important to stress that these ultradian pulses are an intrinsic property of the pituitary–adrenal system, since they occur in response to a constant level of CRH drive. Furthermore, the ultradian period of the pulses (figure 4 and the colour bar in figure 3*a*) is consistent with previous experimental studies, which have reported an interpulse interval range between 47.2 and 54.6 min (Windle *et al*. 1998*a*). Interestingly, our simulations demonstrated that only intermediate values of the CRH drive resulted in ultradian pulses, while high or low CRH drive resulted in a constant response in ACTH and CORT levels (figure 3*a*,*b*,*d*).

**Figure 5.**
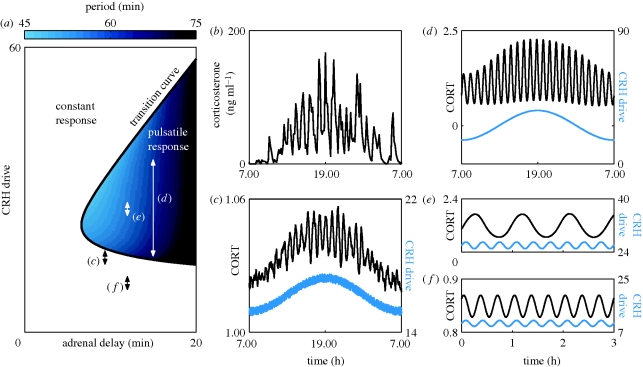
Response of the pituitary–adrenal system to circadian and ultradian patterns of CRH drive. Units of all hormone levels are arbitrary. (*a*) Different combinations of constant CRH drive and delay can lead to two qualitatively different responses. On one side of the transition curve, the pituitary–adrenal system responds with constant levels in ACTH and CORT. On the other side of the transition curve, the pituitary–adrenal system responds with pulsatile fluctuations in the levels of ACTH and CORT, despite the fact that the CRH drive is constant. In the region of pulsatile response, the frequency of the pulses is indicated by the colour bar. (*b*) Experimental data demonstrating an increase in pulse amplitude during the circadian peak. Adapted from Spiga *et al*. (2007). (*c*) Model prediction for a noisy circadian CRH drive close to (but below) the pulsatile region, as indicated by the corresponding arrow in (*a*). Response demonstrates NICOs during the peak of the circadian CRH drive. Computed with a delay of 9.4 min. (*d*) Model prediction for a circadian CRH drive in the pulsatile region, as indicated by the corresponding arrow in (*a*). Response demonstrates increased pulse amplitude during the peak of the circadian CRH drive. Computed with a delay of 15 min. (*e*) Model prediction for ultradian pulses of CRH drive in the pulsatile region, as indicated by the corresponding arrow in (*a*). Response demonstrates a frequency in CORT governed by the pituitary–adrenal system and not by the frequency of the CRH forcing. Computed with a delay of 12 min. (*f*) Model prediction for ultradian pulses of CRH drive in the region of constant response, as indicated by the corresponding arrow in panel (*a*). Response demonstrates a frequency in CORT that is governed by the frequency of the CRH forcing. Computed with a delay of 12 min.

Experimental data demonstrate significant changes in the amplitude of ultradian activity over the course of a 24 hour period (figure 5*b*). Theoretically, we considered the effect of circadian modulation of the PVN by the SCN by driving the pituitary–adrenal system with a circadian (a period of 24 hours) CRH input. Our numerical results parallel experimental observations (Windle *et al*. 1998*b*), whereby the amplitude increases markedly (and the frequency increases slightly) during the high-drive CRH input (figure 5*a*,*d*). Perhaps most significantly, when we included stochastic effects as well as a circadian modulation of the CRH drive, we observed so-called noise-induced coherent oscillations (NICOs) (Wiesenfeld & Moss 1995; Gammaitoni *et al*. 1998) for values of the CRH drive close to (but below) the transition curve (beyond which ultradian pulses were observed in the noise-free scenario). These NICOs closely resembled the experimental data (figure 5*b*,*c*) providing evidence for the hypothesis that feed-forward and feedback interactions within the pituitary–adrenal system are the foundation of ultradian activity observed experimentally (see also figure S8 in the electronic supplementary material for more examples).

We also considered the effect of ultradian CRH pulses on the response of the pituitary–adrenal system. Experimental work has reported a pulsatile pattern of CRH release from the median eminence of the hypothalamus in the rat, with a mean frequency of three pulses per hour (Ixart *et al*. 1991). Our numerical work shows that the pituitary–adrenal system responds to ultradian CRH pulses differently depending on the precise level of these pulses. If their level lies within the region of constant response (figure 5*a*), then the pituitary–adrenal system responds with pulses of CORT at the same frequency as the driving CRH pulses (figure 5*f*). Alternatively, if the level of the CRH pulses lies within the region of pulsatile response (figure 5*a*), then the pituitary–adrenal system responds with pulses of CORT at a frequency governed by the intrinsic properties of the pituitary–adrenal system (figure 5*e*).

Finally, we illustrate how this theoretical approach to understanding the ultradian glucocorticoid rhythm can aid the planning of both experimental and clinical trials. One very important area of clinical medicine that has been linked to both over- and under-activity of the HPA axis is the mood disorders. Depression, in particular, has been consistently associated with significant elevations of HPA activity (Holsboer 2001; Pariante 2003), and many studies have shown that this increased activity is associated with a diminution of sensitivity to the negative feedback by endogenous glucocorticoids. This has been demonstrated by data showing a blunting of endogenous glucocorticoid inhibition following the administration of the synthetic glucocorticoid dexamethasone, or an inhibition of the ACTH response in the dexamethasone-CRH test (Nemeroff 1996; Holsboer 2000; Pariante & Miller 2001; Pariante 2004). Furthermore, glucocorticoid secretion patterns of transgenic mice with reduced GR resemble those patterns seen in subjects with major depression (Pepin *et al*. 1992). Thus, the use of GR antagonists clearly has great potential as a therapeutic strategy in treating patients with mood disorders linked to HPA axis dysfunction.

**Figure 6.**
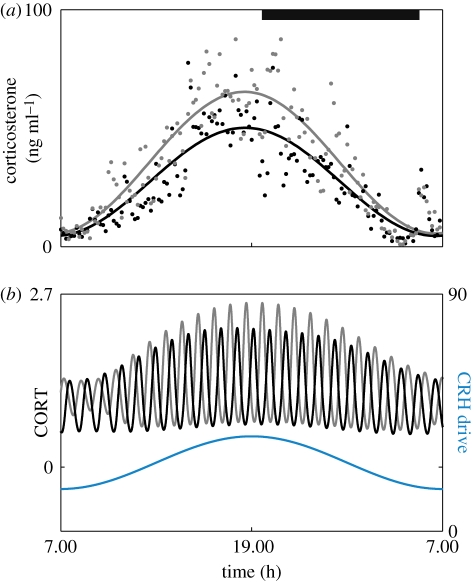
Effect of subchronic treatment with a GR antagonist on the 24 h corticosterone profile. (*a*) Data points represent mean levels of blood corticosterone measured from individual male Sprague–Dawley rats injected twice a day for 5 days with either the GR antagonist Org 34850 (10 mg kg^−1^, subcut., *n* = 7, grey dots) or VEH (5% mulgofen in 0.9% saline, 1 ml kg^−1^, subcut., *n* = 7, black dots). Blood samples were recorded over a 24 h period and collected every 10 min using an automated blood sampling system. Also shown are curves numerically fitted to the two datasets, demonstrating an increase in amplitude during the circadian peak under the influence of Org 34850. Grey bar represents the dark phase (19.15–05.15 h). Adapted from Spiga *et al*. (2007). (*b*) Model simulations show the response of the system to circadian CRH both with (grey) and without (black) a GR antagonist. Infusion of a GR antagonist increases the amplitude of the ultradian glucocorticoid rhythm during the peak of the circadian CRH drive together with a minor increase in ultradian frequency (grey). Computed with a delay of 15 min.

The model we employ here is the first to incorporate the dynamics of the GR in the anterior pituitary (Gupta *et al*. 2007), and therefore provides an ideal platform to investigate the effects that GR antagonists/agonists have on the dynamics of endogenous glucocorticoid secretion. Model results demonstrate that infusion of a GR antagonist (such as Org 34850) increases the amplitude of the ultradian glucocorticoid rhythm during the peak of the circadian CRH drive (figure 6*b*). Furthermore there is a minor increase in ultradian frequency under the influence of a GR antagonist. These theoretical observations are consistent with experimental studies on the rat (Spiga *et al*. 2007), where following 5 days of treatment with the GR antagonist Org 34850, mean corticosterone levels were elevated over the 24 hour cycle (figure 6*a*). Furthermore, this general elevation was the result of an underlying increase in both the amplitude and frequency of the ultradian pulses. In the same study, analysis of the corticosterone rhythm revealed that Org 34850 had its greatest effect during the peak of the circadian rhythm.

Biological systems use rhythmic activity in many time domains, from rapid electrical oscillations in the central nervous system to daily, monthly or even yearly hormone rhythms. Many hormones are also secreted in ultradian patterns, which are important for the maintenance of tissue responsiveness and the avoidance of receptor downregulation. The mechanisms underlying many of these rhythms have been very unclear, and in this paper we have been able to show that relatively simple feed-forward and feedback interactions between the pituitary and adrenal cortex are sufficient to account for the glucocorticoid rhythms we observe experimentally. These oscillations will of course be modified by the gain from the CRH and AVP input to the pituitary, which in turn can be modified by the activity of suprapituitary feedback mediated through both the GR and the mineralocorticoid receptor. This theoretical approach, which simply depends upon systems having delayed feed-forward and feedback pathways, could also provide the basis for understanding ultradian rhythmicity in many other biological systems.
